# Why do we treat adolescent idiopathic scoliosis? What we want to obtain and to avoid for our patients. SOSORT 2005 Consensus paper

**DOI:** 10.1186/1748-7161-1-4

**Published:** 2006-04-10

**Authors:** Stefano Negrini, Theodoros B Grivas, Tomasz Kotwicki, Toru Maruyama, Manuel Rigo, Hans Rudolf Weiss, the members of the Scientific society On Scoliosis Orthopaedic and Rehabilitation Treatment (SOSORT)

**Affiliations:** 1 ISICO (Italian Scientific Spine Institute), Milan, Italy; 2 Orthopaedic Department "Thriasion" General Hospital, Magula, Athens, Greece; 3 University of Medical Sciences, Poznan, Poland; 4 Department of Orthopaedic Surgery, University of Tokyo, Tokyo, Japan; 5 Instituto Èlena Salvá, Barcelona, Spain; 6 Asklepios Katharina Schroth Spinal Deformities Rehabilitation Centre, Bad Sobernheim, Germany; 7 Scientific society On Scoliosis Orthopaedic and Rehabilitation Treatment (SOSORT), Italy

## Abstract

**Background:**

Medicine is a scientific art: once science is not clear, choices are made according to individual and collective beliefs that should be better understood. This is particularly true in a field like adolescent idiopathic scoliosis, where currently does not exist definitive scientific evidence on the efficacy either of conservative or of surgical treatments.

**Aim of the study:**

To verify the philosophical choices on the final outcome of a group of people believing and engaged in a conservative treatment of idiopathic scoliosis.

**Methods:**

We performed a multifaceted study that included a bibliometric analysis, a questionnaire, and a careful Consensus reaching procedure between experts in the conservative treatment of scoliosis (SOSORT members).

**Results:**

The Consensus reaching procedure has shown to be useful: answers changed in a statistically significant way, and 9 new outcome criteria were included. The most important final outcomes were considered Aesthetics (100%), Quality of life and Disability (more than 90%), while more than 80% of preferences went to Back Pain, Psychological well-being, Progression in adulthood, Breathing function, Scoliosis Cobb degrees (radiographic lateral flexion), Needs of further treatments in adulthood.

**Discussion:**

In the literature prevail outcome criteria driven by the contingent treatment needs or the possibility to have measurement systems (even if it seems that usual clinical and radiographic methods are given much more importance than more complex Disability or Quality of Life instruments). SOSORT members give importance to a wide range of outcome criteria, in which clinical and radiographic issues have the lowest importance.

**Conclusion:**

We treat our patients for what they need for their future (Breathing function, Needs of further treatments in adulthood, Progression in adulthood), and their present too (Aesthetics, Disability, Quality of life). Technical matters, such as rib hump or radiographic lateral alignment and rotation, but not lateral flexion, are secondary outcomes and only instrumental to previously reported primary outcomes. We advocate a multidimensional, comprehensive evaluation of scoliosis patients, to gather all necessary data for a complete therapeutic approach, that goes beyond x-rays to reach the person and the family.

## Introduction

Medicine is art: a scientific art, but always art [1,2]. Medicine is art because it implies the ability of a single physician to choose the correct medical means to obtain the right results in front of a specific patient, a person (with his unique characteristics) with a pathology (with his individual expression) [2]. It is a scientific art in the sense that it must be as much related to the literature as possible (evidence based medicine), but conjugated with our everyday experience (evidence based practice) [3-7]. The fact that medicine is art implies that, once science is not clear, choices are made according to individual and collective beliefs, that should be better understood and studied [1,2,4,5,8].

This is particularly true in a field like adolescent idiopathic scoliosis, where currently the lack of RCTs does not allow to have any definitive evidence demonstrating the efficacy either of conservative or of surgical treatments [8-12]. In a remarkable Introduction to their book "Scoliosis: making clinical decisions" [13], Bunch and Patwardhan state: How do you think about scoliosis? Not, 'how do you operate?'; not, 'how do you use orthosis?'; but 'how do you think?'. They continue writing that the model one holds can make a great deal of difference in the treatment plan... mental models clearly determine how we do our work...; models are important primarily because they help us to form a vision with our 'mind's eye', which shapes our own model of disease and treatment and their interaction within the patient [13]. These phrases reasonably well describe why SOSORT members decided to discuss and find a consensus about 'why do we treat?': in a period of time in which in the field of adolescent idiopathic scoliosis it seems that two political parties are facing: one sustaining the need to wait for eventual surgery [10,14-19]; the other, the importance of acting to treat our patients and to avoid more invasive treatments as far as it is possible [8,12,20-25], reasoning on the basis of what we are doing seems to us mandatory. At least, to open a wide and straight discussion with the rest of the world and, in any case, to confront between SOSORT members, that undoubtedly believe in conservative management of scoliosis.

Looking at the thoughts of physicians facing scoliosis, we can see that as far as four centuries B.C. Hyppocrates aimed at restoring the correct anatomy through methods that should act in accordance to nature [26]; it was only after the discovery of X-rays by Roentgen [26] and the consequent understanding of the natural history of scoliosis during growing age; finally it was described by Duval-Beaupere [27], that avoiding progression became a clear cut goal [13,28,29]: these two aims maintain their importance today. But the wide debate on screening for scoliosis, focused on the existence (or not) of efficacy evidence regarding early treatment of scoliosis [12,17,24], opened the Pandora's box of the outcome. This is the reason why we treat patients with adolescent idiopathic scoliosis.

Psychological well-being (PWB) and Quality of Life (QoL), including actual and future Disability (Dis), were already clear in the mind of some pioneers such as Stagnara, who stated that we have to treat human beings with a deformity, not X-rays[29]. Blount and Moe, in the preface to their book 'The Milwaukee Brace', stated the importance of lowering psychological disturbances of braces and even of the help that a well conducted conservative treatment gives to the formation of a mature, adult personality [28]. Along this path, the role of the physician and of the entire treating team is crucial [30]. Today, PWB is considered as determinant [31].

Cobb also considered other outcomes in addition to avoiding the increase (or decrease) of the curve per se: they included improving muscle tone, vital capacity, Aesthetic (Aes) appearance, posture and health, and these were all reasons to prescribe exercises [32].

The cornerstone studies (even if with possible methodological flaws [19]) on the long term natural history of adolescent idiopathic scoliosis published in these years by Weinstein [33-36], the last one with an outstanding editorial by Sponseller [11]. He focused on other issues that could be determinant for patients in the long term, such as Progression in Adulthood (PiA), Breathing Function (BF) (in terms of survival), function in life (marrying, child-bearing, employment, depression, Back Pain [BP], Dis, Aes, neurological impairment). The same issues (function [37,38], QoL[39], marital status and number of children [40], BP [37,38], PiA [37,38], limitations of social activities [39] and sexual function [40]) were considered in long term studies by Danielsson and Nachemson that compared braced and surgically treated idiopathic scoliosis patients to a matched normal sample 20 years after treatment.

In this situation, in which many aims have been proposed it seems that the world of treatment, focused on X-rays and scoliosis Cobb degrees (SCD), is not so strictly conscious of the long term results of which SCD are only a part, and presumably not the most important one;with our paper we aimed to verify the philosophical choices, the final outcome of a group of people believing and engaged in conservative treatment of idiopathic scoliosis.

## Materials and methods

The work has been performed in four distinct parts:

• A systematic literature search on the topic and a bibliometric analysis.

• A questionnaire preparatory to the Consensus Meeting.

• A Consensus reaching procedure, including distribution to participants of results of previous steps (first part of this paper: preliminary document[41]) and a Meeting with a thorough presentation of this document, focused presentations, and long discussions.

• A final Consensus questionnaire.

### Systematic literature search

First, we aimed at identifying all papers that could have faced the philosophical topic of our paper. We searched the Medline database, using free text, from the date of inception to November 2004, without applying any language restriction. We used the words idiopathic scoliosis and set the limits editorial, guideline, meta-analysis and randomized controlled trial. Moreover, we made a hand-search in the Library of an Italian Study Group (Gruppo di Studio della Scoliosi e delle patologie vertebrali) fully dedicated to the rehabilitation of spinal diseases in general and to scoliosis in particular; itcontain more the 300 books on these topics, published from 1978 (creation date of the Study Group) to 2004. According to the abstract, we selected the final papers that were read to search relevant information on the topic to be introduced in the document sent to participants to the Consensus Meeting.

Secondly, we performed a bibliometric analysis: we wanted to verify how many papers that dealt with outcomes in the treatment of adolescent idiopathic scoliosis considered the possibly relevant outcomes. During the preparation of the study, the possibly relevant outcomes of scoliosis treatment have been proposed by the first author and submitted to a preliminary Consensus between the authors of this study: a final list was provided according to Table 1. We searched the Medline database, using free text, from the date of inception to November 2004, without applying any language restriction. We used the words: idiopathic scoliosis that where combined with the operator AND to each single outcome previously identified according to the search strategy reported in Table 2. Together with the bibliometric analysis, we looked at the contents of retrieved paper to add any relevant information on the topic to the document sent to participants to the Consensus Meeting. After the Meeting we performed the same Medline search for the new outcome criteria.

**Table 1:** Pre-Meeting questionnaire. The options listed in the second and third column were required for all outcomes. More free choice Outcomes could be added.

**Outcome ** tick your choice and write your **priority**	**Why ** tick your choice or propose another one	**Type ** tick your choice
Aesthetics	○Literature evidence	○Primary
Breathing function	○General belief	○Secondary
Disability	○Personal belief	○.....
Kypho-lordosis Cobb degrees (radiographic lateral alignment)	○.....	
Needs of further treatments in adulthood		
Back Pain		
Perdriolle degrees (radiographic rotation)		
Progression in adulthood		
Quality of life		
Rib hump		
Scoliosis Cobb degrees (radiographic lateral flexion)		

**Table 2:** Bibliometric analysis: number and percentage of papers in Medline on idiopathic scoliosis related to the outcome considered, and search strategy used.

**Outcome**	**Word used for Medline search (combined with idiopathic scoliosis)**	Papers found	
		**Number**	**Percentage**
Scoliosis Cobb degrees (radiographic lateral flexion)	Cobb	395	16.65%

Back Pain	Pain	207	8.73%
Kypho-lordosis Cobb degrees (radiographic lateral alignment)	Coronal, Lordosis, Kyphosis, Sagittal combined with Cobb, Radiology	197	8.31%
Self control of posture – Sensory motor integration of the corrective ideal pattern	Posture	163	6.87%
Perdriolle degrees (radiographic rotation)	Rotation combined with Cobb, Radiology	156	6.58%

Progression in adulthood	Progression AND Adult, Adulthood	109	4.60%
Balance – Improved processing of vestibular input – Equality of weight bearing	Balance	107	4.51%
Movement of the vertebral column (sagittal plane) – Improved body motor awareness and motor learning skills	Movement	97	4.09%
Breathing function – Exercise efficiency	Breath, Respiration, Ventilation	87	3.67%
Aesthetics	Aesthetics, Appearance, Cosmetic, Cosmesis	85	3.58%
Rib hump	Hump, Prominence, Bunnell (NOT author)	79	3.33%
Psychological well-being	Psychology	72	3.04%
Knowledge and understanding of scoliosis	Knowledge, Belief, Concern	72	3.04%

Quality of life	Quality of life	35	1.48%
Disability	Disability	29	1.22%

### Questionnaire preparatory to the Consensus Meeting

The questionnaire has been prepared through a Consensus between the authors of the study. We made a first version, that was discussed by e-mail in order to produce a second edition that was submitted to a pre-test by e-mail, to obtain the final form. The title of the questionnaire was 'Why do we treat? What we want to obtain and avoid for our patients'and the following questions were proposed:

• When you start a treatment for adolescent idiopathic scoliosis, what do you want to obtain/avoid in adult age for your patient? What are your final **outcomes**? (we propose some possible outcomes: choose yours and eventually propose some new ones)

• Which outcomes are more important (**priority**: 1 high – 2 medium – 3 low)?

• **Why **did you choose these outcomes?

• Define the **type **of outcome: primary or secondary (i.e. an outcome that you strongly want to obtain – primary – could require, to be achieved, the reaching of another outcome – secondary)

The possibly relevant outcomes of scoliosis treatment identified by the authors were listed and the questionnaire was set according to Table 1. Questionnaires constituted the abstracts of the SOSORT Consensus meeting in Milan, January 2005, and have been sent out by e-mail, together with the Preliminary Program, to all the attendees of the 1^st ^International Meeting on Conservative Management of Spinal Deformities held in January 2004 in Barcelona; the Questionnaires have been sent also to all other people interested in the conservative treatment of adolescent idiopathic scoliosis that it was possible to retrieve according to indexed literature. To gather the highest number of opinions, it was required to fill in the questionnaire independently by the participation to the Consensus Meeting, and to reply by e-mail 1.5 months before the Meeting. We received 19 compiled questionnaires.

### Consensus Meeting

A summary document[41], prepared by the authors and containing the results of the questionnaire, was sent out by e-mail to all participants 15 days before the Consensus Meeting to prepare discussion. During the Meeting, in a two-hour session, the results of the Pre-Meeting questionnaire were presented by the first author, two short-presentations were made by participants, and a free-discussion followed. The final questionnaire was prepared and distributed to be compiled by all participants.

### Final questionnaire

According to the results of the Consensus Meeting, a final questionnaire was prepared and proposed to the participants to the Consensus Meeting. The first part was similar to that distributed before the meeting and reported in Table 1: only the question why was avoided, while priority and type of priority were maintained. The final questionnaire was filled-in by 32 participants to the Consensus Meeting in Milan.

### Statistical analysis

All data were managed using Microsoft Excel 2000. Pre and post-Meeting answers were compared using the chi-square test.

## Results

### Literature search

For the purposes of this article we considered 135 papers, while the bibliometric analysis considered a base of 2372 papers published in Medline. We verified that the highest frequency of citation of the considered outcome criteria for adolescent idiopathic scoliosis relate to SCD, while BP, radiographic rotation (Perdriolle degrees – PD) and PiA receive less, but still significant importance. The other identified outcomes are much more less considered: the low importance given to Dis and QoL is particularly noticeable.

### Questionnaire preparatory to the Consensus Meeting

Before the Meeting, BF and PiA were considered by SOSORT members as the most important long term outcome criteria (Table 3); apart from SCD, that in any case ranked 5^th^, the other usual clinical and radiographic measurement were considered of low importance; Dis had the lowest score. BP, Aes, Needs of further Treatments in Adulthood (NTA) and Rib Hump (RH) have been chosen because they are considered important for General belief, while in the other cases for Literature Evidence (Table 4). BP, Kypho-lordosis Cobb degrees (KLD) and PD were the only outcome criteria considered of secondary importance (Table 5). Some responders proposed other outcomes that mainly related to functioning (8 out of 9), in particular to neuro-motorial tasks (6).

**Table 3:** Priorities for each outcome, listed from the highest to the lowest in rank, pre and post Consensus Meeting. The column "Percentage of responders" refers to those that considered each outcome relevant.

**Outcome**		**Rank obtained**		**Percentage of responders**		**Priority**						
						Post			Pre			**Chi**
		Post	Pre	Post	Pre	P1	P2	P3	P1	P2	P3	P
Aesthetics	Aes	1	3	100%	89%	78%	19%	3%	58%	26%	5%	<0.01
Quality of life	QoL	2	4	91%	74%	75%	6%	9%	63%	21%	0%	<0.01
Disability	Dis	3	11	91%	63%	69%	6%	16%	47%	11%	5%	<0.01
Back Pain	BP	4	7	87%	68%	63%	13%	13%	58%	16%	11%	<0.01
Psychological well-being	PWB	5		84%		66%	13%	6%	79%	5%	0%	
Progression in adulthood	PiA	6	2	84%	84%	56%	28%	0%	74%	5%	16%	<0.01
Breathing function	BF	7	1	84%	95%	44%	25%	16%	63%	16%	5%	<0.01
Scoliosis Cobb degrees	SCD	8	5	84%	84%	44%	34%	6%	58%	11%	21%	<0.01
Needs of further treatments in adulthood	NTA	9	6	81%	89%	53%	22%	6%	53%	21%	0%	<0.01
Rib hump	RH	10	8	78%	84%	59%	13%	6%	42%	26%	0%	<0.01
Self control of posture		11		75%		41%	25%	9%				
Perdriolle degrees	PD	12	9	75%	84%	16%	50%	9%	47%	11%	11%	<0.01
Knowledge and understanding of scoliosis		13		72%		44%	16%	13%				
Movement of the vertebral column		14		72%		38%	25%	9%				
Kypho-lordosis Cobb degrees	KLD	15	10	72%	68%	31%	34%	6%	58%	26%	5%	<0.01
Balance		16		69%		44%	19%	6%				
Body motor awareness and learning skills		17		69%		9%	34%	25%				
Sensory motor integration of the corrective pattern		18		62%		41%	16%	6%				
Improved processing of vestibular input		19		59%		6%	31%	22%				
Equality of weight bearing		20		56%		22%	19%	16%				
Exercise efficiency		21		56%		22%	28%	6%				

Post: answers to second questionnaire, proposed after the Consensus Meeting (32 responders) – Pre: answers to first questionnaire, proposed before Consensus Meeting (19 responders) – Chi: Chi-square test

**Table 4:** Motivation for the choice of each outcome, listed according to the median of responses. Pre Consensus Meeting results.

**Outcome**	Motivation of choice		
	**Literature Evidence**	**General belief**	**Personal belief**
Scoliosis Cobb degrees	68%	16%	0%
Breathing function	68%	21%	5%
Progression in adulthood	53%	32%	0%
Quality of life	53%	16%	16%
Disability	47%	11%	11%
Kypho-lordosis Cobb degrees	37%	21%	11%
Perdriolle degrees	37%	16%	16%

Back Pain	42%	16%	32%
Aesthetics	32%	42%	16%
Needs of further treatments in adulthood	32%	21%	37%
Rib hump	32%	21%	37%

**Table 5:** Importance given to each outcome, listed according to the median of responses. Pre and post Consensus Meeting results.

	**Rank obtained**		**Importance**				
			Post		Pre		**Chi**
**Outcome**	Post	Pre	Primary	Secondary	Primary	Secondary	P
Aesthetics	1	4	84%	9%	58%	32%	<0.01
Quality of life	2	2	75%	6%	63%	26%	<0.01
Psychological well-being	3		72%	6%			
Disability	4	7	66%	16%	47%	21%	<0.01
Back Pain	5	9	59%	22%	32%	58%	<0.01
Rib hump	6	3	53%	22%	63%	26%	<0.01
Breathing function	7	6	44%	31%	53%	42%	<0.01
Progression in adulthood	8	1	41%	31%	63%	21%	<0.01
Needs of further treatments in adulthood	9	8	38%	38%	47%	42%	NS
Knowledge and understanding of scoliosis in general and their specific pattern	10		34%	28%			
Balance	11		34%	31%			

Scoliosis Cobb degrees (radiographic lateral flexion)	12	5	28%	44%	53%	32%	<0.05
Self control of posture	13		25%	38%			
Movement of the vertebral column (sagittal plane)	14		22%	38%			
Perdriolle degrees (radiographic rotation)	15	11	19%	50%	21%	53%	<0.01
Kypho-lordosis Cobb degrees (radiographic lateral alignment)	16	10	16%	50%	26%	42%	<0.01
Sensory motor integration of the corrective ideal pattern	17		16%	41%			
Exercise efficiency	18		16%	38%			
Equality of weight bearing	19		13%	31%			
Improved body motor awareness and motor learning skills	20		3%	56%			
Improved processing of vestibular input	21		3%	47%			

Post: answers to second questionnaire, proposed after the Consensus Meeting (32 responders) – Pre: answers to first questionnaire, proposed before Consensus Meeting (19 responders) – Chi: Chi-square test

### Final Consensus

All answers changed in a statistically significant way (Tables 3 and 5), with the exception of the importance given to NTA. The changes do not reflect an increase of Consensus: participants giving the Median value answer decreased from a range of 57.1–93.7% to 47.8–82.7%. The most important final outcomes turned out to be Aes (100%), QoL and Dis (more than 90%), while more than 80% of preferences went to BP, PWB, PiA, BF, SCD, NTA. BP, considered as secondary before the Meeting, became primary in the final Consensus; the opposite happened for SCD, while 3 of the newly included outcomes were considered primary: in particular PWB, but also "Knowledge and understanding of scoliosis in general and their specific pattern" and "Balance". Generally speaking, the importance given to usual clinical and radiographic measurements decreased, while that given to QoL-related issues (including Aes and Dis) increased.

## Discussion

We performed a multifaceted study that included a bibliometric analysis, a questionnaire, and then a careful Consensus reaching procedure between experts in conservative treatment of scoliosis (SOSORT members), to analyse an unusual topic in the literature, such as the motivation for treatment, that is in any case undoubtedly strictly related to everyday clinical behaviours. Looking in general to the results, it seems that in the literature data on radiographic, but also clinical outcome criteria, prevail, while they were considered of the lowest importance in the answers to the questionnaire, and even lower after the Consensus Meeting.

### Aesthetics (Aes)

SOSORT members considered Aes as very relevant and of primary importance, and this increased after the Consensus; only almost 3.5% of the studies in the literature on idiopathic scoliosis relate to this issue. These results presumably reflect the actual absence of means to measure this outcome, (that was encountered by some authors who anyway produced very interesting studies), and the fact of being compelled to rely only on simple observation and individual judgement[42]: there have been some proposals in the literature, including questionnaires[36,39,43-45], and high-tech high-cost instruments [46-50], but none reached any kind of a consensus nor is actually extensively used in everyday clinical practice.

Today, the SRS-22 questionnaire[43,51-53], that includes questions on this topic, even if mainly in a psychological perspective, has been validated in different languages [54-57] and could be useful in the future to have valuable data. Aes is considered when both surgical[42,45,46,58] or conservative[38,59-63] treatment results are reviewed; wearing of braces can give rise to Aes concerns, that have been faced in the literature[62,64]; moreover, the implication of Aes on PWB in scoliosis patients has been thoroughly discussed[44,65,66]. Surgical treatments have been proposed for cosmetic appearance [67-69]. The data on natural history show that idiopathic scoliosis patients with severe curves have some degree of deformity and that they all have cosmetic concerns independently of the importance of the curve[36]; twenty years after treatment, 49% of patients fused and 34% of those braced, (compared to 15% of controls,), showed limitations of social activities mostly due to difficulties with self-consciousness about back appearance, but also to physical limitations and fear of injury, while personal relationships seem not to be influenced[39]; patients feel to look more unattractive in bathing suit (but also wearing clothes) when compared with controls [37-39]. In summary, Aes is a priority one reason to treat our patients: results can be achieved both with bracing and surgery, but also with exercises [20].

### Quality of life (QoL) and Disability (Dis)

QoL is considered of primary and high importance by SOSORT members, with an increase of rank after Consensus, while in the literature this outcome is almost neglected (1.48% of papers). Data on QoL in the literature appear strictly related (sometimes even confused) with data on Dis, but this is not true only for scoliosis; in fact this outcome is almost new in the field of spine research [70] and should be better understood and deepened. To check QoL, disease-specific scales have been applied, like the SRS-22[43,51-53], but also Oswestry and Roland-Morris[37,38,71], (even if these should be regarded mainly as Dis scales, or general health evaluations for children[72] and adults [73-76]); other scales have rarely been used[37-39,72,76]. Anyway, one specific scale, (the QoL Profile for Spine Deformities – QLPSD) appears in the literature[77]. New instruments should be developed, and already well established ones should be used (even if they are usually prepared for adult populations). QoL is another key outcome, and two kind of specific instruments are needed both for children (treatment impact on QoL) and adults (long term results and/or consequences of pathology).

SOSORT members at first considered Dis as the less relevant outcome, in a way reflecting the literature (only 1% of the studies), while after discussion in the Consensus Meeting its importance increased greatly, up to rank 3, as well as the importance. Dis is considered so important by the general community that since 1980 the World Health Organisation, with the aim of better understanding health conditions, proposed a companion classification of the International Classification of Diseases (ICD), named International Classification of Impairment, Disability and Handicap (ICIDH)[78,79]; it became the conceptual basis of the medical specialty of Rehabilitation, and it has recently been totally revised with the new International Classification of Function (ICF) [80-82]: this means that Rehabilitation in general, but also physicians looking at scoliosis patients as persons with an health problem, cannot neglect Dis. It can be measured generally or in a disease-specific way, but only recently a scoliosis specific questionnaire has been developed by SRS [43]. The literature refers to Dis and scoliosis mainly while evaluating BP[71,83-85], because in the field of BP Dis evaluation is a far more established tool. Looking only at deformity, (even if actual and future Dis are well understood as an outcome both by patients and parents[44]), there is very few literature: curiously, brace wearing Dis[77,86,87] is considered, while the immediate effect of being fused is not (at least, we did not find any study); long term studies refer both to previous surgery[37,39,74,75,88] and bracing[38,39]. Patients with severe untreated curves in the long-term reveal some Dis, even if not measured with disease-specific scales[36]. After 20 years, treated patients, both braced and fused, have been considered to have almost the same function as matched controls[37,38], but looking at the data it appears that physical functioning, social activities (more in the surgically-treated than in the braced group) and work (sick-leave) are reduced, while general health only in the operated ones[39]. Dis should be better addressed in the future also by the scoliosis treating community, with more disease-specific scales and looking at short as well as long term results. It is definitively a good reason to treat our patients.

### Back Pain (BP)

Before the Meeting, BP was considered a relevant, but secondary, outcome criterion for SOSORT members, but its priority increased with Consensus and importance became primary. In the literature BP is an important issue associated with idiopathic scoliosis (8.73%, more that any other outcome criterion, apart SCD). Papers are mostly split in two between adulthood (46.8%) and growing age (44%), and relate to almost all possible topics in idiopathic scoliosis treatment. In adults, BP has been reported as follows: life prevalence 61% in high degree curvatures[89], one year prevalence 73%[83], point prevalence 44%[83]; when compared to control groups, the same[90] or higher incidence (44% vs 24%, and 73% vs 56% in one year)[83] was reported. Severity of BP[83,90], persistence or progression with time[90], generalization throughout the back, and radiation into the extremities[83] are higher than in a control group, as well as chronicity (61% vs 35%)[36]. Risk factors include gender (women), pregnancy, fatigue[89], age, degree of scoliotic curvature, lumbar curves[90], smoking[91], and not pre-surgery characteristics, degree of surgical correction, distal level of fusion, degree of Dis[84]. When compared to controls, surgically treated patients have an identical prevalence (73%)[84], but more BP[37], an increased Oswestry score[39], and more degenerative disc changes correlated to lumbar BP intensity[37,92]. Also, braced patients have more BP and disk degeneration than controls[38]. When compared to braced patients, fused ones have a similar Oswestry score[39], but a higher limitation of social activities partially due to BP[39]. Finally, three very interesting papers reported on BP in adolescents [93-95]: point prevalence is between 23% (increased gradually to 58% during follow-up)[93] and 54% [95]. These papers lack a control group, but their results are similar to the actual data on BP in children[96,97]. Risk factors during adolescence are Risser sign, gender, pelvic tilt and bracing [95], particularly progression during brace treatment[94], but not severity of scoliosis[95] or other clinical data[93].

In summary, BP is a good reason to treat our patients on a literature basis looking at adulthood, while, as far as we know today, it is not immediately necessary in adolescents. Presumably, exercises are the best treatment of actual BP, while bracing can have a detrimental effect; conservative treatments seem to have better results on the future than surgery.

### Psychological Well-Being (PWB)

PWB was not included in the first questionnaire, while after Consensus its importance was considered as primary and the priority ranked significantly fifth according to SOSORT members. In the literature it is not so much represented (almost 3%), while it is usually considered in QoL and Dis studies[39,73,76,77,98], and is mostly evaluated with the same instruments used for these outcomes, sometimes with specific sub-scales. Some non systematic reviews have been dedicated to PWB [66,99]. In a large population-based case-control study (34,706 adolescents, 685 with scoliosis) scoliosis showed to be an independent risk factor for suicidal thought, worry and concern over body development, and peer interactions, with gender differences[100]. PWB has been evaluated during brace treatment [64,101,102]: the high negative psychological impact of Milwaukee brace has been pointed out[65,103,104], in particular when compared to TLSOs[77]. A very interesting study analysed PWB and compliance to brace: noncompliant girls did not expect success of treatment, had low self-esteem and did not seek social support, while the contrary was true for noncompliant boys; in short time of brace use, low compliance was best predicted by low reflective thinking and good body-image, and again the contrary was true for patients who had used the brace for >6 months. Curiously, the more the patients experienced sleeping problems, the less they used the brace[105]. PWB predicts satisfaction with final results of surgery[58] and it is used also to evaluate surgery[39,106] and bracing [39,65] in the long term. Scoliosis has been considered as associated with eating disorders, which were not measured[107]. PWB is very important to be evaluated in children as well as in adults: bracing and surgery have a high impact, but also exercises performed for years can have it. While lowering this impact is a key for success, this outcome cannot be neglected in research studies.

### Progression in Adulthood (PiA)

PiA is a rather important primary outcome criterion and is present in as far as 4.6% of papers on idiopathic scoliosis. Its importance has been highly stressed by SOSORT members before the Meeting, while it decreased after Consensus. In the past there was the general belief that after the end of growth there was no progression of scoliosis[33]. Many studies beginning from 1950's begun to show that this was not the case[33,36,89,108]. Data on natural history at 40 years tell us that curves progress in 68% of cases and that it is possible to identify risk factors such as 30° SCD and 33% of radiographic rotation[33]. Obviously PiA correlates with all other outcome criteria and, as far as we know today, it is reasonable to consider this as a primary outcome criterion.

### Needs of further Treatments in Adulthood (NTA)

SOSORT members considered this a quite important primary issue, without big changes after Consensus. We were not able to create a valid Medline search strategy for this outcome criterion, so we lack the data on the literature; anyway, in this respect we could consider those coming from issues that could need a treatment if they appear, such as reduced BF or BP or PiA; the same could be done looking at the data from natural history[36] and long term results of treatment [37-39]. According to those data and our results, NTA seem to be a good reason to treat our patients.

### Breathing Function (BF)

The answers to the questionnaire by SOSORT members gave the highest and primary importance at BF before the Meeting, while Consesus reduced it a lot. In the literature almost 5% of the studies on idiopathic scoliosis considers this issue, that relies on well established, extensively tested and used ways of measurement in respiratory medicine. The outcome "Exercise efficiency", added after Consensus, refers to the same bibliographic references. BF has been used as a means to evaluate patients before and after surgery[109,110], and even the type of surgery to be performed[111], but it has also been evaluated during bracing, both while wearing it [112-115] and in the long term[113]. Many studies focused on exercises and rehabilitation means to improve this function in adolescent idiopathic scoliosis [116-123], while the most related to biomechanics and physiologic studies on pulmonary function in patients[124,125]. Two cornerstone papers have been published by Pehrsson and Nachemson. They found that in the long term mortality for pulmonary deficit is increased with respect to the normal population only in infantile and juvenile scoliosis, not in the adolescent type [126-128], even if subgrouping could have decreased the power of the statistical analysis. In fact, in the 50 year natural history study by Weinstein, patients with severe thoracic curves have a decreased pulmonary function, with an increased risk of shortness of breath[36]. Sponseller raised some questions on the possibility that there may exist a correlation between pulmonary function and mortality (since at least 4 persons in Weinstein's series could have died because of that and the cause for too many others is unknown[11]). Long-term data on braced and surgically treated comparable patients are actually lacking. In summary, BF seems to be a good reason to treat our patients, even if the literature does not fully support this idea (at least for adolescent idiopathic scoliosis and only when looking at mortality data, not when looking at pulmonary function and well-being).

### Radiographic and clinical data

The universally recognized Gold Standard of measurements for scoliosis, SCD to measure radiographic lateral flexion of the spine, ranked first in the literature, with as far as 16.65% of association with idiopathic scoliosis. SOSORT members, that already before the Meeting ranked SCD "only" 5^th ^both for priority and importance (primary); further lowered these results after Consensus, ranking SCD 8^th ^for priority and considering it of secondary importance. Also KLD (more than 8%) and PD (6.58%) have highest ranks for the presence in the literature on idiopathic scoliosis, while they had some of the lowest scores and were of secondary importance for SOSORT members, both before and after the Meeting. On the contrary, a clinical sign as RH has a scant consideration in the literature on idiopathic scoliosis (3,33%); its priority is quite high for SOSORT members and, contrarily to radiographic data, it has been considered a primary outcome both before and after the Meeting. Looking at the literature, it is not possible to go in great details, because papers with these outcomes relate to almost all possible topics in idiopathic scoliosis. Natural history at 40 years tells us that curves under 30° remain stable after the end of growth, while they progress on average of 19° when they are over this value, in particular if they overcome 50°[33]. It seems that being over 30° of lateral flexion and 33% of radiographic rotation is a key risk factor[33]. The hump increased to the actual 36 mm thoracic and 24 mm lumbar in 50 years[36]. While looking at the results of treatment, we do not have results on conservatively or surgically treated populations comparable at start of treatment yet, aside from Danielsson's and Nachemson's paper (Table 6), in which starting data were anyway totally different [37-39]; the results on the sagittal plane in these series are very interesting too. In summary, all radiographic and clinical data that are considered as so important on a literature basis have been rejected in a corner by SOSORT members, excluding the particular cases of SCD (presumably because of the well established literature tradition) and RH (presumably because of its high Aes impact). In fact, we have always to split outcomes in primary and secondary, being the first ones those that are directly perceived by patients and that change their life, and the second ones those that give rise to the former. Looking in this way, all radiographic and clinical data are secondary, because they are clues to possible future (or even actual) BP, Dis, reduced Aes, BF and QoL. Anyway, even if secondary, they for sure are important outcomes, because easily measurable.

**Table 6:** Summary of radiographic and clinical results reported in the three studies by Danielsson and Nachemson.

**Treatment**		**Surgery**	**Brace**	**Controls**
**Years follow-up**		23.3	22.3	
**Population**		146	116	100
**Scoliosis**	*Start*	61.8	33.2	
	*End of treatment*	33.1	29.7	
	*Change from start*	-53.6%	-89.5%	
	*Today*	36.5	37.6	
	*Change from start*	-59.1%	12%	
	*Change from end*	9%	21%	
**Kyphosis**	*Today*	24.5	30.8	38.5
**Lordosis**	*Today*	33.3	44.8	43.9
**Hump**	*Measuring device*	Bunnell	Bunnell	
	*Today*	11.4	10.3	

### The posture, balance and movement related outcomes

All these outcome criteria have been added in the final questionnaire according to first submission and Consensus discussion. The posture-related outcome included "Self control of posture" and "Sensory motor integration of the corrective ideal pattern"; the balance-related outcome included "Balance", "Improved processing of vestibular input" and "Equality of weight bearing"; the movement-related outcome included "Movement of the vertebral column (sagittal plane)" and "Improved body motor awareness and motor learning skills". Aside from "Balance", that was the last one in the primary outcomes, all the others were considered as secondary, while their importance was low (even if chosen by more than 56% of SOSORT members). In the literature 6.9% of papers relates to posture, 4.5% to balance and 4.1% to movement. Posture is the biomechanical representation of a neurological function in which balance and movement are fully included. Posture and balance have been widely considered in studies on aetiology and pathogenesis of scoliosis [129-144], where scoliosis is considered as a neurological disease with a mechanical representation. Studies on these aspects can be split in those mainly neurological[139-141,145-148] and mainly biomechanical, that evaluated standing position[149,150], sitting [151,152], but also gait [153-155] or the relation with backpacks[156,157]. Balance is usually evaluated through force platforms[139-141,150,156-158], while movement requires high-cost complex instruments[153-155,159]. The evaluation of posture is thought to be made through x-rays, not considering that posture is dynamic while x-rays are static: anyway, surface measurements [46-50] are not easily usable in clinics yet; an interesting x-ray approach to posture is comparing supine and standing for SCD [160-162], but also PD[163]. Posture, balance and movement can be increased only with rehabilitation through exercises. Surgery abolishes movement, creates a correct position, but eliminating the dynamic intrinsic to posture, while balance can be impaired. Bracing too impacts negatively on movement and balance, while there could be a positive neurological impact contributing to posture change.

### Cognitive outcome

The outcome "Knowledge and understanding of scoliosis in general and their specific pattern" was added in the final questionnaire according to first submission and Consensus discussion. While it was considered by SOSORT members as a primary outcome, and ranked medium for priority, in the literature only 3% of papers can be connected to this outcome. In spinal rehabilitation the importance of cognitive-behavioural approaches is very well known, mainly in BP. In scoliosis, coping strategies and cognitive-behavioural approaches have not been widely studied, even if there are researches mainly on surgery [164, 165, 166, 167, 168, 169], but also on bracing [105] as well as on exercises [66]. This outcome is important also because it relates to compliance, and should be better studied in the future.

## Conclusion and research recommendation

Why do we treat? What do we want for our patients? In the literature, outcome criteria driven by the contingent treatment needs or the possibility to have measurement systems (even if it seems that usual clinical and radiographic methods are given much more importance than Dis or QoL instruments) prevail: these results could be biased by the method used, that did not include a complete analysis of each single paper, even if the authors' knowledge of the literature and the international Meetings on the topic confirm the idea that we are used to thinking much more to how to do then to why we do[13]. Experts in conservative treatment (SOSORT members) give importance to a wide range of outcome criteria, in which clinical and radiographic issues (apart from SCD, that in any case ranked in a mid position) have the lowest importance. It should be very interesting to propose the same methodology in a sample of high level experts in surgical care, to verify the answers to the same questions. Today, research recommendations should be made to develop valid, reliable and possibly low-cost instruments to evaluate Aes, PWB, posture, balance and movement, while existing QoL and Dis scales should be improved. Moreover, on the basis of our results, we advocate a multidimensional, comprehensive evaluation of scoliosis patients, to gather all necessary data for a complete therapeutic approach, that goes beyond x-rays to reach the person and the family.

## References

[B1] Lawn B (1996). L'arte perduta di guarire.

[B2] Sacks O (1985). L'uomo che scambiò sua moglie per un cappello.

[B3] Fowler PB (1995). Evidence-based medicine. Lancet.

[B4] Grol R, Grimshaw J (2003). From best evidence to best practice: effective implementation of change in patients' care. Lancet.

[B5] Malterud K (2001). The art and science of clinical knowledge: evidence beyond measures and numbers. Lancet.

[B6] Naylor CD (1995). Grey zones of clinical practice: some limits to evidence-based medicine. Lancet.

[B7] White KL (1995). Evidence-based medicine. Lancet.

[B8] Negrini S, Brambilla C, Carabalona R (2004). Social Acceptability of Treatments for Adolescent Idiopathic Scoliosis. Pediatr Rehabil.

[B9] Bridwell KH (1999). Surgical treatment of idiopathic adolescent scoliosis. Spine.

[B10] Dickson RA (1999). Spinal deformity--adolescent idiopathic scoliosis. Nonoperative treatment. Spine.

[B11] Sponseller PD (2003). Sizing up scoliosis. Jama.

[B12] Negrini S, Aulisa L, Ferraro C, Fraschini P, Masiero S, Simonazzi P, Tedeschi C, Venturin A (2005). Italian guidelines on rehabilitation treatment of adolescents with scoliosis or other spinal deformities. Eura Medicophys.

[B13] Bunch WH, Patwardhan AG (1989). Scoliosis Making Clinical Decisionsed.

[B14] Dickson RA (1989). Idiopathic scoliosis. Bmj.

[B15] Dickson RA (1984). Screening for scoliosis. Br Med J (Clin Res Ed).

[B16] Dickson RA, Weinstein SL (1999). Bracing (and screening)--yes or no?. J Bone Joint Surg Br.

[B17] Goldberg CJ, Dowling FE, Hall JE, Emans JB (1993). A statistical comparison between natural history of idiopathic scoliosis and brace treatment in skeletally immature adolescent girls. Spine.

[B18] Goldberg CJ, Dowling FE, Fogarty EE, Moore DP (1995). School scoliosis screening and the United States Preventive Services Task Force. An examination of long-term results. Spine.

[B19] Hawes MC (2003). Health and function of patients with untreated idiopathic scoliosis. Jama.

[B20] Hawes MC (2003). The use of exercises in the treatment of scoliosis: an evidence-based critical review of the literature. Pediatr Rehabil.

[B21] Negrini S, Antonini G, Carabalona R, Minozzi S (2003). Physical exercises as a treatment for adolescent idiopathic scoliosis. A systematic review. Pediatr Rehabil.

[B22] Rigo M, Reiter C, Weiss HR (2003). Effect of conservative management on the prevalence of surgery in patients with adolescent idiopathic scoliosis. Pediatr Rehabil.

[B23] Weiss HR, Weiss G, Schaar HJ (2002). Conservative management in patients with scoliosis--does it reduce the incidence of surgery?. Stud Health Technol Inform.

[B24] Winter RB, Lonstein JE (1997). To brace or not to brace: the true value of school screening. Spine.

[B25] Weiss HR, Weiss G, Schaar HJ (2003). Incidence of surgery in conservatively treated patients with scoliosis. Pediatr Rehabil.

[B26] Marketos SG, Skiadas P (1999). Hippocrates. The father of spine surgery. Spine.

[B27] Duval-Beaupere G, Lamireau T (1985). Scoliosis at less than 30 degrees. Properties of the evolutivity (risk of progression). Spine.

[B28] Blount WP, Moe JH (1973). The Milwaukee Brace.

[B29] Stagnara P (1976). Les deformations du rachis.

[B30] Osmond-Clarke H, Blount WP and Moe JH (1973). Scoliosis. The Milwaukee Brace.

[B31] Hall JE (1997). Controversial issues in spinal deformity surgery. J Pediatr Orthop.

[B32] Riseborough EJ, Herndon JH (1985). Scoliosis and other deformities of the axial skeleton.

[B33] Weinstein SL (1999). Natural history. Spine.

[B34] Weinstein SL (2000). Bristol-Myers Squibb/Zimmer award for distinguished achievement in orthopaedic research. Long-term follow-up of pediatric orthopaedic conditions. Natural history and outcomes of treatment. J Bone Joint Surg Am.

[B35] Weinstein SL (1986). Idiopathic scoliosis. Natural history. Spine.

[B36] Weinstein SL, Dolan LA, Spratt KF, Peterson KK, Spoonamore MJ, Ponseti IV (2003). Health and function of patients with untreated idiopathic scoliosis: a 50-year natural history study. Jama.

[B37] Danielsson AJ, Nachemson AL (2003). Back pain and function 23 years after fusion for adolescent idiopathic scoliosis: a case-control study-part II. Spine.

[B38] Danielsson AJ, Nachemson AL (2003). Back pain and function 22 years after brace treatment for adolescent idiopathic scoliosis: a case-control study-part I. Spine.

[B39] Danielsson AJ, Wiklund I, Pehrsson K, Nachemson AL (2001). Health-related quality of life in patients with adolescent idiopathic scoliosis: a matched follow-up at least 20 years after treatment with brace or surgery. Eur Spine J.

[B40] Danielsson AJ, Nachemson AL (2001). Childbearing, curve progression, and sexual function in women 22 years after treatment for adolescent idiopathic scoliosis: a case-control study. Spine.

[B41] Negrini S, Grivas T, Kotwicki T, Maruyama T, Rigo M, Weiss HR, Members of the Study group On Scoliosis Orthopaedic and Rehabilitation Treatment (SOSORT) Why we treat adolescent idiopathic scoliosis? What we want to obtain and to avoid for our patients. SOSORT 2005 Consensus Paper – Topic 3. http://www.isico.it.

[B42] Buchanan R, Birch JG, Morton AA, Browne RH (2003). Do you see what I see? Looking at scoliosis surgical outcomes through orthopedists' eyes. Spine.

[B43] Asher M, Min Lai S, Burton D, Manna B (2003). The reliability and concurrent validity of the scoliosis research society-22 patient questionnaire for idiopathic scoliosis. Spine.

[B44] Bridwell KH, Shufflebarger HL, Lenke LG, Lowe TG, Betz RR, Bassett GS (2000). Parents' and patients' preferences and concerns in idiopathic adolescent scoliosis: a cross-sectional preoperative analysis. Spine.

[B45] Sanders JO, Polly DWJ, Cats-Baril W, Jones J, Lenke LG, O'Brien MF, Stephens Richards B, Sucato DJ (2003). Analysis of patient and parent assessment of deformity in idiopathic scoliosis using the walter reed visual assessment scale. Spine.

[B46] Hackenberg L, Hierholzer E, Potzl W, Gotze C, Liljenqvist U (2003). Rasterstereographic back shape analysis in idiopathic scoliosis after anterior correction and fusion. Clin Biomech (Bristol, Avon).

[B47] Liu XC, Thometz JG, Lyon RM, Klein J (2001). Functional classification of patients with idiopathic scoliosis assessed by the Quantec system: a discriminant functional analysis to determine patient curve magnitude. Spine.

[B48] Theologis TN, Jefferson RJ, Simpson AH, Turner-Smith AR, Fairbank JC (1993). Quantifying the cosmetic defect of adolescent idiopathic scoliosis. Spine.

[B49] Weisz I, Jefferson RJ, Carr AJ, Turner-Smith AR, McInerney A, Houghton GR (1989). Back shape in brace treatment of idiopathic scoliosis. Clin Orthop Relat Res.

[B50] Weisz I, Jefferson RJ, Turner-Smith AR, Houghton GR, Harris JD (1988). ISIS scanning: a useful assessment technique in the management of scoliosis. Spine.

[B51] Asher M, Lai SM, Burton D, Manna B (2002). Trunk deformity correction stability following posterior instrumentation and arthrodesis for idiopathic scoliosis. Stud Health Technol Inform.

[B52] Asher M, Min Lai S, Burton D, Manna B (2003). Discrimination validity of the scoliosis research society-22 patient questionnaire: relationship to idiopathic scoliosis curve pattern and curve size. Spine.

[B53] Asher M, Min Lai S, Burton D, Manna B (2003). Scoliosis research society-22 patient questionnaire: responsiveness to change associated with surgical treatment. Spine.

[B54] Alanay A, Cil A, Berk H, Acaroglu RE, Yazici M, Akcali O, Kosay C, Genc Y, Surat A (2005). Reliability and validity of adapted Turkish Version of Scoliosis Research Society-22 (SRS-22) questionnaire. Spine.

[B55] Monticone M, Carabalona R, Negrini S (2004). Reliability of the Scoliosis Research Society-22 Patient Questionnaire (Italian version) in mild adolescent vertebral deformities. Eura Medicophys.

[B56] Climent JM, Bago J, Ey A, Perez-Grueso FJ, Izquierdo E (2005). Validity of the Spanish version of the Scoliosis Research Society-22 (SRS-22) Patient Questionnaire. Spine.

[B57] Bago J, Climent JM, Ey A, Perez-Grueso FJ, Izquierdo E (2004). The Spanish version of the SRS-22 patient questionnaire for idiopathic scoliosis: transcultural adaptation and reliability analysis. Spine.

[B58] Koch KD, Buchanan R, Birch JG, Morton AA, Gatchel RJ, Browne RH (2001). Adolescents undergoing surgery for idiopathic scoliosis: how physical and psychological characteristics relate to patient satisfaction with the cosmetic result. Spine.

[B59] Gabos PG, Bojescul JA, Bowen JR, Keeler K, Rich L (2004). Long-term follow-up of female patients with idiopathic scoliosis treated with the Wilmington orthosis. J Bone Joint Surg Am.

[B60] Grivas TB, Vasiliadis E, Chatziargiropoulos T, Polyzois VD, Gatos K (2003). The effect of a modified Boston brace with anti-rotatory blades on the progression of curves in idiopathic scoliosis: aetiologic implications. Pediatr Rehabil.

[B61] Rigo M (2003). Radiological and cosmetic improvement 2 years after brace weaning--a case report. Pediatr Rehabil.

[B62] Veldhuizen AG, Cheung J, Bulthuis GJ, Nijenbanning G (2002). A new orthotic device in the non-operative treatment of idiopathic scoliosis. Med Eng Phys.

[B63] Willers U, Normelli H, Aaro S, Svensson O, Hedlund R (1993). Long-term results of Boston brace treatment on vertebral rotation in idiopathic scoliosis. Spine.

[B64] Korovessis P, Stamatakis M, Baikousis A, Kirkos C, Kavouris A (1996). Vertical transmission of the hip rolls due to wearing of TLSO for scoliosis. J Spinal Disord.

[B65] Noonan KJ, Dolan LA, Jacobson WC, Weinstein SL (1997). Long-term psychosocial characteristics of patients treated for idiopathic scoliosis. J Pediatr Orthop.

[B66] Reichel D, Schanz J (2003). Developmental psychological aspects of scoliosis treatment. Pediatr Rehabil.

[B67] Barrett DS, MacLean JG, Bettany J, Ransford AO, Edgar MA (1993). Costoplasty in adolescent idiopathic scoliosis. Objective results in 55 patients. J Bone Joint Surg Br.

[B68] Grivas TB (2002). Surgery is performed for cosmetic reasons. Stud Health Technol Inform.

[B69] Owen R, Turner A, Bamforth JS, Taylor JF, Jones RS (1986). Costectomy as the first stage of surgery for scoliosis. J Bone Joint Surg Br.

[B70] Wood-Dauphinee SL (2001). Assessment of back-related quality of life: the continuing challenge. Spine.

[B71] Niemeyer T, Bovingloh AS, Grieb S, Schaefer J, Halm H, Kluba T (2005). Low back pain after spinal fusion and Harrington instrumentation for idiopathic scoliosis. Int Orthop.

[B72] Ugwonali OF, Lomas G, Choe JC, Hyman JE, Lee FY, Vitale MG, Roye DPJ (2004). Effect of bracing on the quality of life of adolescents with idiopathic scoliosis. Spine J.

[B73] Gotze C, Liljenqvist UR, Slomka A, Gotze HG, Steinbeck J (2002). Quality of life and back pain: outcome 16.7 years after Harrington instrumentation. Spine.

[B74] Padua R, Ceccarelli E, Aulisa AG, Pitta L, Aulisa L (2002). Outcome of Harrington surgery for idiopathic scoliosis. SF-36 and Roland questionnaires assessment. Stud Health Technol Inform.

[B75] Padua R, Padua S, Aulisa L, Ceccarelli E, Padua L, Romanini E, Zanoli G, Campi A (2001). Patient outcomes after Harrington instrumentation for idiopathic scoliosis: a 15- to 28-year evaluation. Spine.

[B76] Freidel K, Petermann F, Reichel D, Steiner A, Warschburger P, Weiss HR (2002). Quality of life in women with idiopathic scoliosis. Spine.

[B77] Climent JM, Sanchez J (1999). Impact of the type of brace on the quality of life of Adolescents with Spine Deformities. Spine.

[B78] Halbertsma J, Heerkens YF, Hirs WM, de Kleijn-de Vrankrijker MW, Dorine Van Ravensberg CD, Napel HT (2000). Towards a new ICIDH. International Classification of Impairments, Disabilities and Handicaps. Disabil Rehabil.

[B79] Post MW, de Witte LP, Schrijvers AJ (1999). Quality of life and the ICIDH: towards an integrated conceptual model for rehabilitation outcomes research. Clin Rehabil.

[B80] Stucki G, Ewert T, Cieza A (2003). Value and application of the ICF in rehabilitation medicine. Disabil Rehabil.

[B81] Stucki G, Ewert T, Cieza A (2002). Value and application of the ICF in rehabilitation medicine. Disabil Rehabil.

[B82] Stucki G, Grimby G (2004). Applying the ICF in medicine. J Rehabil Med.

[B83] Mayo NE, Goldberg MS, Poitras B, Scott S, Hanley J (1994). The Ste-Justine Adolescent Idiopathic Scoliosis Cohort Study. Part III: Back pain. Spine.

[B84] Poitras B, Mayo NE, Goldberg MS, Scott S, Hanley J (1994). The Ste-Justine Adolescent Idiopathic Scoliosis Cohort Study. Part IV: Surgical correction and back pain. Spine.

[B85] Shapiro GS, Taira G, Boachie-Adjei O (2003). Results of surgical treatment of adult idiopathic scoliosis with low back pain and spinal stenosis: a study of long-term clinical radiographic outcomes. Spine.

[B86] Nicholson GP, Ferguson-Pell MW, Smith K, Edgar M, Morley T (2002). Quantitative measurement of spinal brace use and compliance in the treatment of adolescent idiopathic scoliosis. Stud Health Technol Inform.

[B87] Nicholson GP, Ferguson-Pell MW, Smith K, Edgar M, Morley T (2003). The objective measurement of spinal orthosis use for the treatment of adolescent idiopathic scoliosis. Spine.

[B88] Pratt RK, Burwell RG, Cole AA, Webb JK (2002). Patient and parental perception of adolescent idiopathic scoliosis before and after surgery in comparison with surface and radiographic measurements. Spine.

[B89] Ascani E, Bartolozzi P, Logroscino CA, Marchetti PG, Ponte A, Savini R, Travaglini F, Binazzi R, Di Silvestre M (1986). Natural history of untreated idiopathic scoliosis after skeletal maturity. Spine.

[B90] Jackson RP, Simmons EH, Stripinis D (1983). Incidence and severity of back pain in adult idiopathic scoliosis. Spine.

[B91] Scott SC, Goldberg MS, Mayo NE, Stock SR, Poitras B (1999). The association between cigarette smoking and back pain in adults. Spine.

[B92] Danielsson AJ, Cederlund CG, Ekholm S, Nachemson AL (2001). The prevalence of disc aging and back pain after fusion extending into the lower lumbar spine. A matched MR study twenty-five years after surgery for adolescent idiopathic scoliosis. Acta Radiol.

[B93] Ramirez N, Johnston CE, Browne RH (1997). The prevalence of back pain in children who have idiopathic scoliosis. J Bone Joint Surg Am.

[B94] Ramirez N, Johnston CE, Browne RH, Vazquez S (1999). Back pain during orthotic treatment of idiopathic scoliosis. J Pediatr Orthop.

[B95] Joncas J, Labelle H, Poitras B, Duhaime M, Rivard CH, Le Blanc R (1996). [Dorso-lumbal pain and idiopathic scoliosis in adolescence]. Ann Chir.

[B96] Balague F, Dudler J, Nordin M (2003). Low-back pain in children. Lancet.

[B97] Balague F, Troussier B, Salminen JJ (1999). Non-specific low back pain in children and adolescents: risk factors. Eur Spine J.

[B98] Freidel K, Reichel D, Steiner A, Warschburger P, Petermann F, Weiss HR (2002). Idiopathic scoliosis and quality of life. Stud Health Technol Inform.

[B99] Eliason MJ, Richman LC (1984). Psychological effects of idiopathic adolescent scoliosis. J Dev Behav Pediatr.

[B100] Payne WK, Ogilvie JW, Resnick MD, Kane RL, Transfeldt EE, Blum RW (1997). Does scoliosis have a psychological impact and does gender make a difference?. Spine.

[B101] Andersen MO, Andersen GR, Thomsen K, Christensen SB (2002). Early weaning might reduce the psychological strain of Boston bracing: a study of 136 patients with adolescent idiopathic scoliosis at 3.5 years after termination of brace treatment. J Pediatr Orthop B.

[B102] MacLean WEJ, Green NE, Pierre CB, Ray DC (1989). Stress and coping with scoliosis: psychological effects on adolescents and their families. J Pediatr Orthop.

[B103] Fallstrom K, Cochran T, Nachemson A (1986). Long-term effects on personality development in patients with adolescent idiopathic scoliosis. Influence of type of treatment. Spine.

[B104] Kahanovitz N, Snow B, Pinter I (1984). The comparative results of psychologic testing in scoliosis patients treated with electrical stimulation or bracing. Spine.

[B105] Lindeman M, Behm K (1999). Cognitive strategies and self-esteem as predictors of brace-wear noncompliance in patients with idiopathic scoliosis and kyphosis. J Pediatr Orthop.

[B106] Orvomaa E (1998). Psychological evaluations of patients operated for idiopathic scoliosis by the Harrington method. Int J Rehabil Res.

[B107] Smith FM, Latchford G, Hall RM, Millner PA, Dickson RA (2002). Indications of disordered eating behaviour in adolescent patients with idiopathic scoliosis. J Bone Joint Surg Br.

[B108] Edgar MA, Mehta MH (1988). Long-term follow-up of fused and unfused idiopathic scoliosis. J Bone Joint Surg Br.

[B109] Lenke LG, White DK, Kemp JS, Bridwell KH, Blanke KM, Engsberg JR (2002). Evaluation of ventilatory efficiency during exercise in patients with idiopathic scoliosis undergoing spinal fusion. Spine.

[B110] Lindh M, Bjure J (1975). Lung volumes in scoliosis before and after correction by the Harrington instrumentation method. Acta Orthop Scand.

[B111] Kovac V, Puljiz A, Smerdelj M, Pecina M (2001). Scoliosis curve correction, thoracic volume changes, and thoracic diameters in scoliotic patients after anterior and after posterior instrumentation. Int Orthop.

[B112] Ferrari K, Goti P, Sanna A, Misuri G, Gigliotti F, Duranti R, Iandelli I, Ceppatelli S, Scano G (1997). Short-term effects of bracing on exercise performance in mild idiopathic thoracic scoliosis. Lung.

[B113] Korovessis P, Filos KS, Georgopoulos D (1996). Long-term alterations of respiratory function in adolescents wearing a brace for idiopathic scoliosis. Spine.

[B114] Sevastikoglou JA, Linderholm H, Lindgren U (1976). Effect of the Milwaukee brace on vital and ventilatory capacity of scoliotic patients. Acta Orthop Scand.

[B115] Margonato V, Fronte F, Rainero G, Merati G, Veicsteinas A (2005). Effects of short term cast wearing on respiratory and cardiac responses to submaximal and maximal exercise in adolescents with idiopathic scoliosis. Eura Medicophys.

[B116] DiRocco P (1981). Cardiopulmonary effects of scoliosis. Am Correct Ther J.

[B117] DiRocco PJ, Breed AL, Carlin JI, Reddan WG (1983). Physical work capacity in adolescent patients with mild idiopathic scoliosis. Arch Phys Med Rehabil.

[B118] DiRocco PJ, Vaccaro P (1988). Cardiopulmonary functioning in adolescent patients with mild idiopathic scoliosis. Arch Phys Med Rehabil.

[B119] Durmala J, Dobosiewicz K, Jendrzejek H, Pius W (2002). Exercise efficiency of girls with idiopathic scoliosis based on the ventilatory anaerobic threshold. Stud Health Technol Inform.

[B120] Hawes MC (2001). Improved chest expansion in idiopathic scoliosis. Psychosom Med.

[B121] Hawes MC, Brooks WJ (2002). Reversal of the signs and symptoms of moderately severe idiopathic scoliosis in response to physical methods. Stud Health Technol Inform.

[B122] Hawes MC, Brooks WJ (2001). Improved chest expansion in idiopathic scoliosis after intensive, multiple-modality, nonsurgical treatment in an adult. Chest.

[B123] Smyth RJ, Chapman KR, Wright TA, Crawford JS, Rebuck AS (1986). Ventilatory patterns during hypoxia, hypercapnia, and exercise in adolescents with mild scoliosis. Pediatrics.

[B124] Kotani T, Minami S, Takahashi K, Isobe K, Nakata Y, Takaso M, Inoue M, Maruta T, Akazawa T, Ueda T, Moriya H (2004). An analysis of chest wall and diaphragm motions in patients with idiopathic scoliosis using dynamic breathing MRI. Spine.

[B125] Leong JC, Lu WW, Luk KD, Karlberg EM (1999). Kinematics of the chest cage and spine during breathing in healthy individuals and in patients with adolescent idiopathic scoliosis. Spine.

[B126] Pehrsson K, Bake B, Larsson S, Nachemson A (1991). Lung function in adult idiopathic scoliosis: a 20 year follow up. Thorax.

[B127] Pehrsson K, Danielsson A, Nachemson A (2001). Pulmonary function in adolescent idiopathic scoliosis: a 25 year follow up after surgery or start of brace treatment. Thorax.

[B128] Pehrsson K, Nachemson A, Olofson J, Strom K, Larsson S (1992). Respiratory failure in scoliosis and other thoracic deformities. A survey of patients with home oxygen or ventilator therapy in Sweden. Spine.

[B129] Cheung KM, Wang T, Poon AM, Carl A, Tranmer B, Hu Y, Luk KD, Leong JC (2005). The effect of pinealectomy on scoliosis development in young nonhuman primates. Spine.

[B130] Machida M, Saito M, Dubousset J, Yamada T, Kimura J, Shibasaki K (2005). Pathological mechanism of idiopathic scoliosis: experimental scoliosis in pinealectomized rats. Eur Spine J.

[B131] Machida M, Dubousset J, Satoh T, Murai I, Wood KB, Yamada T, Ryu J (2001). Pathologic mechanism of experimental scoliosis in pinealectomized chickens. Spine.

[B132] Machida M (1999). Cause of idiopathic scoliosis. Spine.

[B133] Machida M, Murai I, Miyashita Y, Dubousset J, Yamada T, Kimura J (1999). Pathogenesis of idiopathic scoliosis. Experimental study in rats. Spine.

[B134] Machida M, Miyashita Y, Murai I, Dubousset J, Yamada T, Kimura J (1997). Role of serotonin for scoliotic deformity in pinealectomized chicken. Spine.

[B135] Machida M, Dubousset J, Imamura Y, Miyashita Y, Yamada T, Kimura J (1996). Melatonin. A possible role in pathogenesis of adolescent idiopathic scoliosis. Spine.

[B136] Pompeiano O, Manzoni D, Miele F (2002). Pineal gland hormone and idiopathic scoliosis: possible effect of melatonin on sleep-related postural mechanisms. Arch Ital Biol.

[B137] Dubousset J, Wicart P, Pomero V, Barois A, Estournet B (2002). [Thoracic scoliosis: exothoracic and endothoracic deformations and the spinal penetration index]. Rev Chir Orthop Reparatrice Appar Mot.

[B138] Dubousset J (2001). Scoliosis and its pathophysiology: do we understand it?. Spine.

[B139] Byl NN, Holland S, Jurek A, Hu SS (1997). Postural imbalance and vibratory sensitivity in patients with idiopathic scoliosis: implications for treatment. J Orthop Sports Phys Ther.

[B140] Byl NN, Gray JM (1993). Complex balance reactions in different sensory conditions: adolescents with and without idiopathic scoliosis. J Orthop Res.

[B141] Adler N, Bleck EE, Rinsky LA, Young W (1986). Balance reactions and eye-hand coordination in idiopathic scoliosis. J Orthop Res.

[B142] Burwell RG, Dangerfield PH (2002). Etiologic theories of idiopathic scoliosis: neurodevelopmental concepts to be evaluated. Stud Health Technol Inform.

[B143] Burwell RG (2003). Aetiology of idiopathic scoliosis: current concepts. Pediatr Rehabil.

[B144] Burwell RG, Cole AA, Cook TA, Grivas TB, Kiel AW, Moulton A, Thirlwall AS, Upadhyay SS, Webb JK, Wemyss-Holden SA (1992). Pathogenesis of idiopathic scoliosis. The Nottingham concept. Acta Orthop Belg.

[B145] Lu WW, Hu Y, Luk KD, Cheung KM, Leong JC (2002). Paraspinal muscle activities of patients with scoliosis after spine fusion: an electromyographic study. Spine.

[B146] Cheung J, Sluiter WJ, Veldhuizen AG, Cool JC, Van Horn JR (2002). Perception of vertical and horizontal orientation in children with scoliosis. J Orthop Res.

[B147] Cheung J, Veldhuizen AG, Halbertsma JP, Maurits NM, Sluiter WJ, Cool JC, Van Horn JR (2004). The relation between electromyography and growth velocity of the spine in the evaluation of curve progression in idiopathic scoliosis. Spine.

[B148] Cheung J, Halbertsma JP, Veldhuizen AG, Sluiter WJ, Maurits NM, Cool JC, van Horn JR (2005). A preliminary study on electromyographic analysis of the paraspinal musculature in idiopathic scoliosis. Eur Spine J.

[B149] Zabjek KF, Leroux MA, Coillard C, Rivard CH, Prince F (2005). Evaluation of segmental postural characteristics during quiet standing in control and Idiopathic Scoliosis patients. Clin Biomech (Bristol, Avon).

[B150] Nault ML, Allard P, Hinse S, Le Blanc R, Caron O, Labelle H, Sadeghi H (2002). Relations between standing stability and body posture parameters in adolescent idiopathic scoliosis. Spine.

[B151] Bennett BC, Abel MF, Granata KP (2004). Seated postural control in adolescents with idiopathic scoliosis. Spine.

[B152] Gram MC, Hasan Z (1999). The spinal curve in standing and sitting postures in children with idiopathic scoliosis. Spine.

[B153] Frigo C, Carabalona R, Dalla Mura M, Negrini S (2003). The upper body segmental movements during walking by young females. Clin Biomech (Bristol, Avon).

[B154] Kramers-de Quervain IA, Muller R, Stacoff A, Grob D, Stussi E (2004). Gait analysis in patients with idiopathic scoliosis. Eur Spine J.

[B155] Giakas G, Baltzopoulos V, Dangerfield PH, Dorgan JC, Dalmira S (1996). Comparison of gait patterns between healthy and scoliotic patients using time and frequency domain analysis of ground reaction forces. Spine.

[B156] Chow DH, Kwok ML, Cheng JC, Lao ML, Holmes AD, Au-Yang A, Yao FY, Wong MS (2005). The effect of backpack weight on the standing posture and balance of schoolgirls with adolescent idiopathic scoliosis and normal controls. Gait Posture.

[B157] Chow DH, Kwok ML, Au-Yang AC, Holmes AD, Cheng JC, Yao FY, Wong MS (2005). The effect of load carriage on the gait of girls with adolescent idiopathic scoliosis and normal controls. Med Eng Phys.

[B158] Allard P, Chavet P, Barbier F, Gatto L, Labelle H, Sadeghi H (2004). Effect of body morphology on standing balance in adolescent idiopathic scoliosis. Am J Phys Med Rehabil.

[B159] Rahmatalla A, Chockalingam N, Dangerfield P, Ahmed el N, Cochrane T, Dove J, Maffulli N (2002). Movement analysis of scoliotic subjects using Fastrak. Stud Health Technol Inform.

[B160] Duval-Beaupere G, Lespargot A, Grossiord A (1985). Flexibility of scoliosis. What does it mean? Is this terminology appropriate?. Spine.

[B161] Duval-Beaupere G (1992). Rib hump and supine angle as prognostic factors for mild scoliosis. Spine.

[B162] Duval-Beaupere G (1996). Threshold values for supine and standing Cobb angles and rib hump measurements: prognostic factors for scoliosis. Eur Spine J.

[B163] Yazici M, Acaroglu ER, Alanay A, Deviren V, Cila A, Surat A (2001). Measurement of vertebral rotation in standing versus supine position in adolescent idiopathic scoliosis. J Pediatr Orthop.

[B164] LaMontagne LL, Hepworth JT, Cohen F, Salisbury MH (2004). Adolescent scoliosis: effects of corrective surgery, cognitive-behavioral interventions, and age on activity outcomes. Appl Nurs Res.

[B165] LaMontagne LL, Hepworth JT, Cohen F, Salisbury MH (2004). Adolescents' coping with surgery for scoliosis: effects on recovery outcomes over time. Res Nurs Health.

[B166] LaMontagne L, Hepworth JT, Salisbury MH, Cohen F (2003). Effects of coping instruction in reducing young adolescents' pain after major spinal surgery. Orthop Nurs.

[B167] Lamontagne LL, Hepworth JT, Salisbury MH, Riley LP (2003). Optimism, anxiety, and coping in parents of children hospitalized for spinal surgery. Appl Nurs Res.

[B168] LaMontagne LL, Hepworth JT, Cohen F, Salisbury MH (2003). Cognitive-behavioral intervention effects on adolescents' anxiety and pain following spinal fusion surgery. Nurs Res.

[B169] Lamontagne LL, Hepworth JT, Salisbury MH (2001). Anxiety and postoperative pain in children who undergo major orthopedic surgery. Appl Nurs Res.

